# Bayesian model selection maps for group studies

**DOI:** 10.1016/j.neuroimage.2009.08.051

**Published:** 2010-01-01

**Authors:** M.J. Rosa, S. Bestmann, L. Harrison, W. Penny

**Affiliations:** a Wellcome Trust Centre for Neuroimaging, UCL Institute of Neurology, University College London, 12 Queen Square, WC1N 3BG, UK; b Sobell Department of Motor Neuroscience and Movement Disorders, UCL Institute of Neurology, University College London, 33 Queen Square, WC1N 3BG, UK; c York Neuroimaging Centre, University of York, YO10 5DG, York, UK

## Abstract

This technical note describes the construction of posterior probability maps (PPMs) for Bayesian model selection (BMS) at the group level. This technique allows neuroimagers to make inferences about regionally specific effects using imaging data from a group of subjects. These effects are characterised using Bayesian model comparisons that are analogous to the *F*-tests used in statistical parametric mapping, with the advantage that the models to be compared do not need to be nested. Additionally, an arbitrary number of models can be compared together. This note describes the integration of the Bayesian mapping approach with a random effects analysis model for BMS using group data. We illustrate the method using fMRI data from a group of subjects performing a target detection task.

## Introduction

Given a set of candidate hypotheses, or models, scientists can use Bayesian inference to update their beliefs about the respective hypotheses, in light of new experimental data. The most likely hypothesis can then be identified using Bayesian model selection (BMS).

BMS is based on the model evidence, i.e., the probability of obtaining observed data, *y*, given model *m*, *p*(*y*|*m*). In a group study, one obtains a separate evidence value for each model and for each subject. Under the assumption that the data are independent from subject to subject, these evidence values can be multiplied together to produce a single evidence value for each model. The ratio of resulting model evidences then forms what is known as the group Bayes factor (Stephan and Penny, 2007).

In more recent work, Stephan et al. (2009) have shown that the group Bayes factor approach corresponds to what is more generally known as a fixed effects analysis (Penny and Holmes, 2006). The fixed effects (FFX) approach can be understood from a generative model perspective in which a vector of values *r* correspond to the frequencies of models used in the population at large. FFX then assigns a model, drawn using *r*, to be used by all members of the group. A drawback of the FFX approach is that it does not account for between-subject variability which can make the resulting inferences over-confident. Additionally, it is not robust to the presence of outliers.

Stephan et al. (2009) contrast the FFX approach with a proposed random effects (RFX) approach, in which a (potentially different) model is assigned to each member of the group. Stephan et al. (2009) then describe Bayesian estimation procedures for obtaining the posterior distribution *p*(*r*|*Y*), where *Y* comprises data from all subjects. Contrary to the FFX approach, this method correctly takes into account the variability between subjects and is also robust to outliers.

In earlier work, Penny et al. (2007) have developed Bayesian spatiotemporal models for fMRI data, which provide within-subject model evidence maps. Voxel-wise comparison of these maps allows neuroimagers to make inferences about regionally specific effects. These comparisons are analogous to the *F*-tests used in statistical parametric mapping (Friston et al., 2007), with the advantage that the models to be compared do not need to be nested. Additionally, an arbitrary number of models can be compared together.

The Bayesian approach is useful when there is no natural nesting of hypotheses. A trend in recent neuroimaging research, for example, is to fit computational models to behavioural data, and then to use variables from these data fits as regressors in general linear models of fMRI data (Montague et al., 2004; Behrens et al., 2008). A natural extension of this approach is to derive different sets of regressors from different computational models, and so allow fMRI to provide evidence in favour of one model or another. An example in the field of behavioural control would be to compare different models of ‘value updating’ (e.g., the Rescorla–Wagner model versus the ‘temporal difference’ model (Montague et al., 2004)).

In this technical note, we describe the combination of the mapping approach for providing log-evidence maps for each model and subject, with the RFX approach described in Stephan et al. (2009). This procedure constructs posterior probability maps (PPMs) for BMS inference at the group level. We illustrate the method using fMRI data from a group of subjects performing a cued two-choice reaction time task and compare it with a FFX analysis of the same data.

The note is structured as follows. In the next section, we briefly revisit the model evidence. We then describe the commonly used FFX approach, and the recently developed RFX approach for BMS at the group level. We then proceed to describe how BMS maps can be constructed from previously estimated log-evidence maps and, in the Results section, apply this method to fMRI group data from a target detection task.

## Theory

### Model evidence

The model evidence, *p*(*y*|*m*), is the probability of obtaining observed data, *y*, given model, *m*, and is at the heart of Bayesian model selection (BMS). In general, the model evidence is not straightforward to compute, since this computation involves integrating out the dependency on the model parameters, *θ*:

(1) p(y|m)=∫p(y|θ,m)p(θ|m)dθ

Sampling or iterative analytic methods can be used to approximate the above integral. A common technique used in neuroimaging is the variational Bayes (VB) approach (Penny et al., 2003). This is an analytic method that can be formulated by analogy with statistical physics as a gradient ascent on the “negative free energy,” *F*(*m*), of the system. In other words, the aim of VB is to maximise *F*(*m*) with respect to a variational density, or approximate posterior density *q*(*θ*), maximising a lower bound on the logarithm of the model evidence (log-model evidence) (Beal, 2003):

(2) logp(y|m)=F(m)+KL(q(θ)||p(θ|y,m)).

The last term in Eq. (2) is the Kullback–Leibler (KL) divergence between the approximate posterior density, *q*(*θ*), and the true posterior, *p*(*θ*|*y*, *m*). This quantity is always positive, or zero when the densities are identical, and therefore log *p*(*y*|*m*) is bounded below by *F*(*m*). By iterative optimisation, the KL divergence is minimised and *F*(*m*) becomes an increasingly tighter lower bound on the desired log-model evidence. Under the assumption that this bound is tight, BMS can then proceed using *F*(*m*) as a surrogate for the log-model evidence.

The variational Free Energy is but one approximation to the model evidence, albeit one that is widely used in neuroimaging (Woolrich et al., 2004a; Sato et al., 2004). Other approximations include the computationally more expensive Annealed Importance Sampling (AIS) method (Beal and Ghahramani, 2003), and the simpler but potentially less accurate Bayesian Information Criterion (BIC) and Akaike Information Criterion (AIC) measures (Penny et al., 2004). In extensive simulations of graphical model structures, Beal and Ghahramani (2003) found that the variational approach outperformed BIC, at relatively little extra computational cost, and approached the performance of AIS, but with much less computational cost.

### Bayesian model selection

The ratio of model evidences is known as the Bayes factor (BF). Given uniform priors over models, the posterior model probability is greater than 0.95 if the BF is greater than 20. Bayes factors have also been stratified into different ranges deemed to correspond to different strengths of evidence. ‘Strong’ evidence, for example, corresponds to a BF of over 20 (Kass and Raftery, 1995). In a group study, one obtains a separate model evidence value for each model *k* and for each subject *n*. The following sections describe two different approaches for model inference at the group level.

#### Fixed effects

**Fig. 1:**
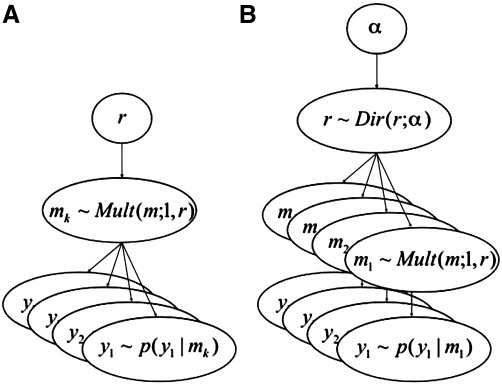
Graphical models underlying (A) fixed and (B) random effects inference on model space at the group level. FFX assigns a model, drawn using *r*, to be used by all members of the group, while for RFX, a (potentially different) model is assigned to each member of the group. Mult(*m*;1, *r*) corresponds to Mult(*m*; N, *r*), when the number of observations *N* is equal to 1. See the main text for a detailed explanation of the two different inference approaches.

Until very recently, most group studies have adopted what is known as the group Bayes factor (GBF) approach (Stephan and Penny, 2007). The GBF can be obtained by simply multiplying the individual BFs for all *N* subjects (assuming subjects are independent):

(3) $$\begin{array}{l} {\text{GBF}}_{i,j}=\prod_{n=1}^{N}{\text{BF}}_{i,j}^{\left(n\right)}\\ \text{log}{\text{GBF}}_{i,j}=\sum_{n=1}^{N}\text{log}p\left(y_{n}|m_{ni}\right)-\sum_{n=1}^{N}\text{log}p\left(y_{n}|m_{nj}\right), \end{array}$$

where the subscripts *i* and *j* denote the *i*-th and *j*-th models being compared. The log GBF is therefore simply the difference of the model evidences aggregated over subjects. Although this is a straightforward method for model selection and has been used in a number of neuroimaging studies (Summerfield and Koechlin, 2008; Stephan et al., 2007), Stephan et al. (2009) have recently shown that the group Bayes factor approach corresponds to what is more generally known as a fixed effects (FFX) analysis. The FFX approach can be understood from a generative model perspective in which a probability vector, *r* = [*r*_1_, ..., *r*_*K*_], with 0 ≤ *r*_*k*_ ≤ 1 and $\sum_{k=1}^{K}r_{k}=1$, represents frequencies of models used in the population at large. FFX then assigns a model (from the *K* models considered), drawn using *r*, to be used by all members of the group (Fig. 1A). This approach, as is the case with FFX approaches based on effect size (Penny and Holmes, 2006), does not therefore correctly take into account between-subject variability.

#### Random effects

In contrast to the FFX approach, Stephan et al. (2009) have developed a hierarchical model for making inferences on the posterior density of the model frequencies themselves, *p*(*r*|*Y*), given the data from all subjects, *Y*. This method can be viewed as a random effects (RFX) approach, in which a (potentially different) model is assigned to each member of the group (Fig. 1B). In other words, the assignment of different models to subjects is treated as a random process. The corresponding random variables are drawn from a density, *p*(*r*|*α*), which then defines a distribution on how likely it is that model *k* generated the data for subject *n*, *p*(*m*_*nk*_  = 1) = *r*_*k*_, where *m*_*nk*_ ∈ {0, 1} and $\sum_{k=1}^{K}m_{nk}=1$. Because, for each subject, this latter distribution has a multinomial form (i.e., each subject uses either model *k* = 1, 2, ..., *K*), it is natural to choose *p*(*r*|*α*) as a Dirichlet density, as the Dirichlet is conjugate to the multinomial (Bernardo and Smith, 2001). The parameters of this Dirichlet, *α* = [*α*_1_, ..., *α*_*K*_], are related to the unobserved ‘occurrences’ of the models in the population.

The same authors then describe an estimation procedure to invert this hierarchical model and estimate the posterior distribution over *r*. Briefly, this optimisation scheme begins by assuming that each model has been ‘observed’ once, *α*_0_ = [1, ..., 1], and proceeds by updating estimates of *α* until convergence. The following pseudo-code schematizes this iterative procedure and the quantities computed at each step:

(4) $$\begin{array}{l} \alpha =\alpha_{0}\\ \text{until convergence}\\ \;{\text{compute g}}_{nk}\\ \;\text{compute }\beta \\ \;\text{update }\alpha =\alpha_{0}+\beta \\ \text{end}\text{.} \end{array}$$

In the first step, the normalised posterior belief that model *k* generated the data from subject *n*, *g*_*nk*_, is computed using the following equations:

(5) $$\begin{array}{l} u_{nk}=\text{exp}\left(\text{log}\;p\left(y_{n}|m_{nk}\right)+\Psi \left(\alpha_{k}\right)-\Psi \left(\alpha_{S}\right)\right)\\ u_{n}=\sum_{k=1}^{K}u_{nk}\\ g_{nk}=\frac{u_{nk}}{u_{n}}, \end{array}$$

where log *p*(*y*_*n*_|*m*_*nk*_) is the log-model evidence from subject *n* and model *k*, *Ψ* is the digamma function, *Ψ*(*α*_*k*_) = ∂logΓ(*α*_*k*_) / ∂*α*_*k*_, and *α*_*S*_ = Σ_*k*_*α*_*k*_. For the results in this paper, we use the variational free energy approximation to the model evidence, as described in Penny and Flandin (2007). In the next step, the expected number of subjects whose data are believed to have been generated by model *k* is computed for all models:

(6) $$\beta_{k}=\sum_{n}g_{nk}.$$

Finally, using the result from the previous step, the *α* parameters are updated (Eq. (4)).

After optimisation, the posterior distribution *p*(*r*|*Y*; *α*) can be used for model inference at the group level. One can, for instance, use this distribution to compute the expected multinomial parameters, 〈*r*_*k*_〉, which encode the expected posterior probability of model *k* being selected for a randomly chosen subject:

(7) $$\langle r_{k}\rangle =\alpha_{k}/\left(\alpha_{1}+...+\alpha_{K}\right),$$

Another option is to use *p*(*r*|*Y*; *α*) to compute an exceedance probability, *φ*_*k*_, which corresponds to the belief that model *k* is more likely than any other (of the *K* models compared), given the data from all subjects:

(8) $$\varphi_{k}=p\left(\prod_{j\neq k}r_{k}>r_{j}|Y;\alpha \right).$$

Exceedance probabilities are particularly intuitive when comparing just two models (see, for example, Fig. 6B) as they can be written:

(9) $$\varphi_{1}=p\left(r_{1}>r_{2}|Y;\alpha \right)=p\left(r_{1}>0.5|Y;\alpha \right).$$

In the next section, we describe how this approach can be applied voxel-wise to previously obtained log-evidence maps, in order to construct posterior probability maps and exceedance probability maps for Bayesian inference at the group level.

### Bayesian model selection maps

#### Within-subject maps

In an earlier work, Penny et al. (2005) developed a Bayesian spatiotemporal model for fMRI data, which allows inferences to be made about regionally specific effects using posterior probability maps (PPMs). Similar approaches have been developed previously by Hartvig and Jensen (2000) and Woolrich et al. (2004b). PPMs represent images of the probability that a contrast of parameter estimates exceeds some specified threshold and their construction has previously been described in Friston and Penny (2003).

The model developed by Penny et al. (2005) extends previous Bayesian modelling approaches for fMRI (Friston et al., 2002a,b) by, among other things, introducing a spatial prior on the regression coefficients. This prior embodies the knowledge that activations are spatially contiguous and results in an ability to detect more subtle activations. Although this spatial prior was initially two-dimensional (limited to voxels contained in the same slice), this work has since been extended to three-dimensional priors (Harrison et al., 2008).

In more recent work, Penny et al. (2007) have shown how the model evidence can be used to construct within-subject PPMs for model selection. As compared to model comparison based on *F*-tests using classical inference, this approach has the advantage of allowing the comparison of non-nested models. Additionally, it allows for the simultaneous comparison of an arbitrary number of models. As compared to earlier work (Friston and Penny, 2003) based on PPMs of effect size, the approach is advantageous in not requiring an effect size threshold.

In this technical note, we have combined the mapping approach used in Penny et al. (2007) to provide log-evidence maps for each model and subject, with the RFX approach described in Stephan et al. (2009) in order to produce group maps for model selection.

#### Group maps

Once the log-evidence maps have been estimated for each subject and model, as described above, it is possible to construct between-subject posterior probability maps that enable inference on model space at the group level. These maps are created by applying the RFX approach described above at every voxel, *i*, of the log-evidence data, which produces a family of posterior distributions, *p*(*r*_*ki*_|*Y*_*i*_). We can then construct the PPMs for each model *k* by plotting the posterior expectation, 〈*r*_*ki*_|*Y*_*i*_〉 for every voxel *i* (Eq. (7)) at which the value exceeds a user-specified threshold, *γ*.

In addition to the group-level PPMs, the RFX approach also allows the construction of exceedance probability maps (EPMs). These constitute an exceedance probability for each voxel *i*, *φ*_*ki*_ (see Eq. (8)) and for each model *k*. Again, these maps are thresholded at a user-specified value *γ*.

**Fig. 2:**
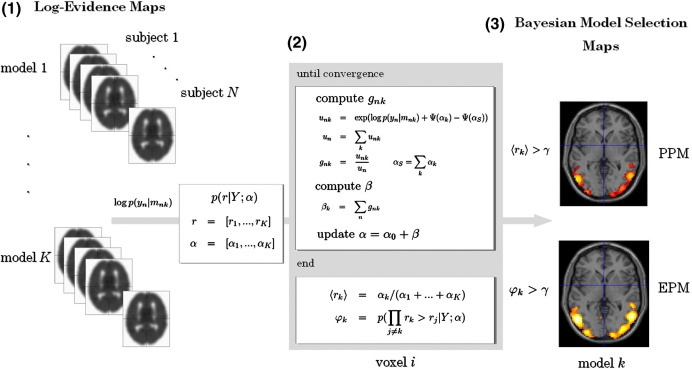
Schematic representation of the method for constructing Bayesian model selection (BMS) maps for group studies. (1) The first step involves estimating log-evidence maps for each subject and model. (2) The RFX approach for BMS described in the text is then applied in a voxel-wise manner to the log-evidence data. (3) The BMS maps (posterior probability map, PPM; exceedance probability map, EPM) for each model are then constructed by plotting the posterior and exceedance probabilities at each voxel (〈*r*_*ki*_〉 and *φ_ki_*, respectively), using a threshold, *γ*, to visualise the resulting image. See the main text for a detailed explanation of the different steps involved in this procedure.

The maps described here can be constructed as whole-brain images or images from selected regions of interest. The latter can be created by specifying a mask image, which limits the construction of the maps to voxels contained in the mask. Such masks can be created, for example, using a functional localiser analysis (Friston et al., 2006). The overall approach for creating BMS maps for group studies is shown in Fig. 2.

It is also possible to create group maps using an FFX rather than the above RFX approach. This is implemented simply by summing the log-evidence images over subjects for each model (see Eq (3)). Posterior model probabilities are then obtained by exponentiating the resulting sums and normalising to unity.

## Results

In this section, we illustrate the application of our method to fMRI data acquired from subjects performing a simple Posner-type cued target detection task. Imaging data were recorded using a Siemens VISION system (Siemens, Erlangen, Germany) operating at 2 T. A total of 330 functional volumes (28 slices) were recorded for each subject, using T2⁎-weighted MRI transverse echo-planar images (EPI) (64 × 64 matrix, 3 × 3 × 5 mm^3^ voxel size, TE = 40 ms) with blood oxygenation level dependent (BOLD) contrast. Effective repetition time (TR) per volume was 2.15 s.

Imaging data were preprocessed using Statistical Parametric Mapping (SPM5, Wellcome Trust Centre for Neuroimaging, http://www.fil.ion.ucl.ac.uk/spm/) implemented in Matlab 6 (The Mathworks Inc., USA). Functional volumes were realigned and unwarped (Andersson et al., 2001), and the resulting volumes were normalised to a standard EPI template based on the Montreal Neurological Institute (MNI) reference brain in Talairach space (Talairach and Tournoux, 1988) and resampled to 3 × 3 × 3 mm voxels. The time series in each voxel were high pass filtered at 1/128 Hz to remove low frequency confounds and scaled to a grand mean of 100 over voxels and scans within each session.

Twelve subjects responded to a right- or left-sided target (“+ O” or “O +”) appearing for 250 ms on a screen by spatially compatible button presses using the right and left index finger, respectively. The target was preceded by a visuospatial cue (“< + <” or “> + >”) presented for 250 ms and appearing 1000 ms before the target. Four different event types were presented randomly: validly cued right and left button presses (66 trials each), and invalidly cued right and left button presses (17 trials each). During null events (165 trials), the central fixation cross was maintained with no presentation of cue or target, and no corresponding button press. The intertrial interval was 2000 ms. Responses were recorded by computer using COGENT Cognitive Interface Software (Wellcome Trust Centre for Neuroimaging, London, UK).

### Nested models

To construct the BMS maps described above, we began by specifying two different models for the acquired fMRI data.

First, we specified a ‘Validity’ model (model 1), including a column of 1's for the session mean and additional regressors for validly and invalidly cued trials. These two regressors were parametrically modulated by reaction times. Second, we specified a ‘Null’ model (model 2) comprising a single column for the session mean. Comparison of these two models could therefore be implemented using a standard *F*-test approach with classical SPMs, because model 2 is nested within model 1. More generally, however, the BMS approach does not require the models to be nested (see below).

Each model was estimated with SPM5, using the first-level Bayesian estimation procedure described in Penny et al. (2005). This produced a voxel-wise whole-brain log-model evidence map for every subject and model estimated (see left panel of Fig. 2). These maps were then smoothed with an 8 mm half width Gaussian kernel.

We then applied the RFX approach described above to the group model evidence data in a voxel-wise manner. This procedure yielded a posterior probability map (PPM) and exceedance probability map (EPM) for each model. In addition, we compared these PPMs with those obtained using a FFX analysis.

**Fig. 3:**
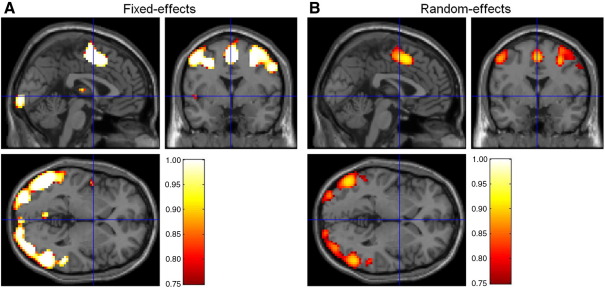
Group-level PPMs for the ‘Validity’ model from (A) fixed and (B) random effects analysis. The maps therefore show brain regions encoding cue validity. These maps were thresholded to show regions where the posterior model probability of the ‘Validity’ model is greater than *γ* = 0.75. The FFX approach does not account for between-subject variability and, consequently, can appear over-confident.

Fig. 3 shows the group-level PPMs for the ‘Validity’ model (model 1) constructed using the FFX (A) and RFX (B) method, and thresholded in order to show the brain regions where the posterior probability for model 1 is above *γ* = 0.75.

**Fig. 4:**
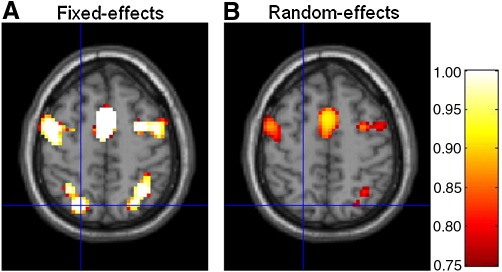
Group-level PPMs (*z* = 59 mm, Talairach coordinates) for the ‘Validity’ model from (A) fixed and (B) random effects analysis. The maps were thresholded to show regions where the posterior probability of the ‘Validity’ model is greater than *γ* = 0.75. The position of the crossbars (Talairach coordinates: [− 21, − 73, 59] mm) indicates a cluster that is only visible for the FFX maps, suggesting that this approach may be over-confident.

These regions show strong evidence in favour of the ‘Validity’ model. More specifically, these regions comprise brain areas one would a priori expect to be generally involved in a Posner-type task as used in the example data set presented here (Rounis et al., 2006), including motor areas (peak voxel Talairach coordinates [*x*, *y*, *z*] in millimeters: left supplementary motor area [0, 5, 56], right precentral gyrus [33, − 4, 53], and left precentral gyrus [− 51, − 4, 56]) as well as visual- and attention-related regions (Talairach coordinates [*x*, *y*, *z*] in millimeters: right inferior temporal gyrus [57, − 67, 2], left inferior temporal gyrus [− 51, − 76, 2], and left middle temporal gyrus [− 54, − 73, 5]). Fig. 3 shows that the FFX and RFX approaches for inference on model space yielded similar results. However, because the FFX approach does not accommodate between-subject variability the resulting inferences are somewhat over-confident. This is also illustrated in Fig. 4 where, for example, the position of the crossbars indicates a cluster that is only visible for the FFX maps.

**Fig. 5:**
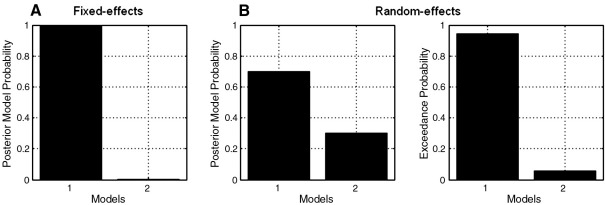
Posterior model probabilities obtained by comparing the ‘Validity’ and ‘Null’ model (models 1 and 2, respectively) at an example voxel, [− 21, − 73, 59] mm (Talairach coordinates), using a (A) fixed and (B) random effects analysis. For the RFX analysis, we include the exceedance probabilities at the same voxel. As can be seen, the RFX analysis produces lower posterior probabilities for model 1 than does the FFX approach.

The probabilities obtained for both models at the peak voxel of this cluster are shown in Fig. 5. As can be seen, the RFX analysis produces lower posterior probabilities for model 1 than does the FFX approach. Moreover, this probability is approximately 0.7 (Fig. 5B), which is slightly below the threshold, *γ* = 0.75, used for constructing the maps in Fig. 4. For this reason the corresponding cluster is missing in the RFX map (Fig. 4B).

**Fig. 6:**
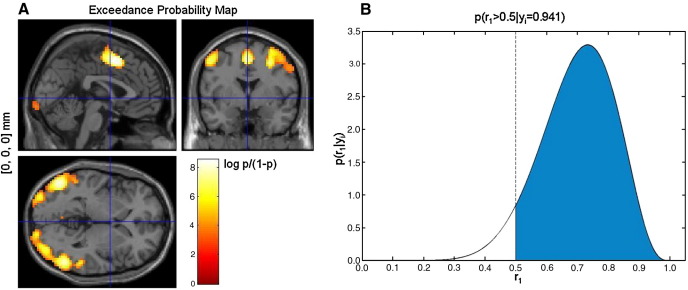
(A) Group-level exceedance probability map (EPM) (log-odds scale) for the ‘Validity’ model. The map was thresholded to show regions where the exceedance probability for the ‘Validity’ model is greater than *γ* = 0.95. (B) Posterior distribution and exceedance probability for the same model at an example voxel, [− 21, − 73, 59] mm (Talairach coordinates).

Fig. 6A plots the exceedance probability map (EPM) for the ‘Validity’ model using a threshold of *γ* = 0.95. For this model, the exceedance probability is given by *φ*_*i*__1 _= *p*(*r*_*i*__1_ > 0.5) and Fig. 6A plots *φ*_*i*__1_ only at those voxels for which *φ*_*i*__1_ > *γ*. This map is similar to the PPM shown in Fig. 3B, which plots 〈*r*_*i*__1_〉 at those voxels for which 〈*r*_*i*__1_〉 > *γ*.

To better illustrate what is being plotted in Fig. 6A, we have plotted the posterior distribution for the same model, *p*(*r*_1_|*Y*), obtained at one example voxel (Fig. 6B). The shaded region corresponds to *r*_1_ > 0.5 and for this voxel encompasses 94.1% of the total mass of the posterior distribution. Therefore, the exceedance probability value plotted for this voxel is 0.941.

**Fig. 7:**
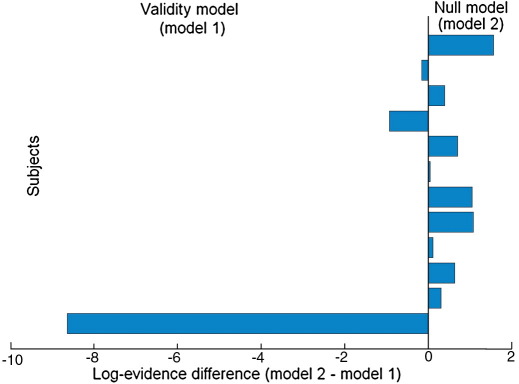
Log-model evidence differences between the ‘Null’ and ‘Validity’ models (model 2 and model 1, respectively) at voxel [− 29, 0, 49] mm (Talairach coordinates), for the 12 subjects analysed. The data clearly show that one subject (bottom row) is an outlier.

Stephan et al. (2009) have noted that the RFX approach is more robust in the presence of outliers than is the FFX method. We examined this in our data by inspecting regions in the BMS maps showing contradictory results for FFX and RFX. Consequently, we found groups of voxels at which model 1 was clearly the best model for the FFX analysis and model 2 for the RFX. We then looked at the log-model evidence values for all subjects at these voxels and found that the reason for the discrepancy was indeed an outlying subject. Fig. 7 shows an example of this, where almost all subjects indicate that model 2 is best, except for a single outlying subject with an extreme evidence value favouring model 1.

**Fig. 8:**
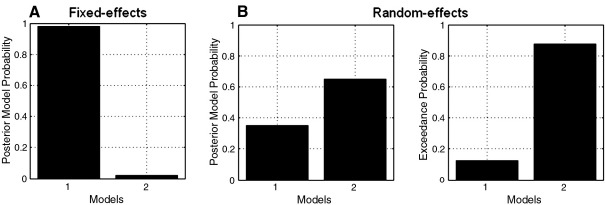
Posterior model probabilities obtained by comparing the ‘Validity’ and ‘Null’ model (models 1 and 2, respectively) at voxel [− 29, 0, 49] mm (Talairach coordinates), using a (A) fixed and (B) random effects analysis. For the RFX analysis, we include the exceedance probabilities at the same voxel. The voxel chosen here belongs to a brain region where FFX and RFX analyses yield different results due to the presence of an outlier (see Fig. 7).

**Fig. 9:**
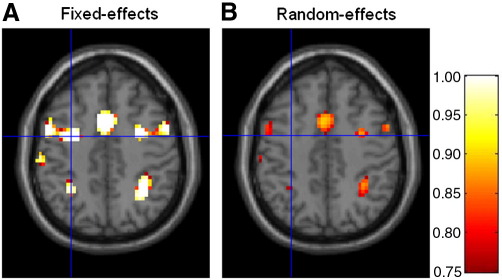
Group-level PPMs (slice *z* = 49 mm, Talairach coordinates) for the ‘Validity’ model from (A) fixed and (B) random effects analysis. The maps were thresholded to show regions where the posterior model probability of the ‘Validity’ model is greater than *γ* = 0.75. The crossbars indicate a cluster of voxels where one of the subjects is clearly an outlier (Fig. 7).

The posterior probabilities obtained for this voxel (for which one of the subjects is an outlier) reveal that the FFX results are in favour of the ‘Validity’ model, while RFX suggests that the ‘Null’ model is better (Figs. 8A and B), as can also be seen in the respective PPMs (Fig. 9). Moreover, the exceedance probability value for the ‘Null’ model is almost 80%, which indicates strong evidence in favour of model 2 at this voxel.

These results corroborate Stephan et al. (2009) who have also shown that the RFX approach is more robust in the presence of outliers.

### Non-nested models

The BMS approach presented here is particularly suited for comparing non-nested models. Here, we use the aforementioned example dataset to illustrate how BMS can be applied to compare models for which there is no natural nesting.

In principle, there is no upper bound on the number of models to be compared; however, for the purpose of this technical note, we focus on two alternative non-nested models. Previous work has shown that the history of past events in an experimental task can be formalized using information theory (Strange et al., 2005; Harrison et al., 2006), under ideal observer assumptions. One finding was that activity in a widespread frontoparietal network, including bilateral fusiform, parietal, lateral and medial premotor and inferior frontal regions, as well as in bilateral thalamus relates to the surprise conveyed by a trial event. This activation pattern is similar to the task-related activity shown by our ‘Validity’ model. The ‘surprise’ inherent in an event (e.g., an infrequently occurring invalidly cued trial) is based on the probability of that event, given previous trials. Here, we calculated surprise from posterior probabilities updated on a trial-by-trial basis using Bayes rule (see Strange et al. (2005) and Mars et al. (2008) for further details). This was then used to predict neuronal responses measured in our fMRI experiment. More specifically, we modeled the onsets of trials with a stick function that was parametrically modulated by the surprise on a given trial. We refer to this model as the ‘Ideal Observer’ model.

Alternatively, one can relax the assumption that participants are ideal observers. One could, for example, compare a number of models in which the duration and rate of decay with which past observations (trials) are weighted are differently parameterized. For illustrating the BMS approach, we here focus on one case only, in which only a window of data comprising the four most recent trials was taken into account for computing surprise (see Bestmann et al. (2008) for details). We refer to this model as the ‘Window’ model. This model is suboptimal from an information theoretic perspective because the observer fails to properly accumulate the evidence available within a block. However, as the brain also has other criteria to optimise (e.g., energy use, speed of response), it could be that imaging data provide evidence for it.

Each of the above models was estimated using the first-level Bayesian estimation procedure, as described above, producing voxel-wise whole-brain log-model evidence maps for every subject and model estimated. These maps were then smoothed with an 8 mm half width Gaussian kernel.

**Fig. 10:**
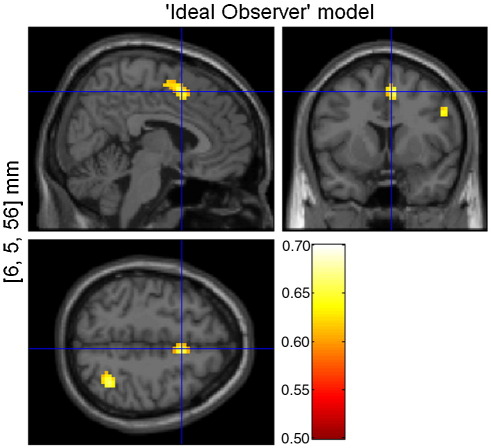
Group-level PPM for the ‘Ideal Observer’ model from random effects analysis. The map is thresholded to show regions where the posterior model probability of the ‘Ideal Observer’ model is greater than *γ* = 0.6.

Fig. 10 shows the group-level PPM for the two locations in which the posterior model probability for the ‘Ideal Observer’ model is greater than *γ* = 0.6. We focused explicitly on task-related brain regions, as identified in the group-level PPM for the ‘Validity’ model (see Fig. 3B). Our BMS suggests that activity in these two regions (Talairach coordinates [*x*, *y*, *z*] in millimeters: supplementary motor area [6, 5, 56] and right superior parietal lobule [36, − 58, 59]) is best explained by the surprise conveyed by an event, as estimated by an ideal observer.

## Discussion

In this note, we have presented the construction of posterior probability maps allowing for Bayesian model selection at the group level. These maps are produced by combining a model evidence mapping approach with an RFX approach for model selection.

We have illustrated our method by applying it to fMRI data from a group study and compared the resulting maps with those obtained using a FFX analysis. As expected, both analyses yielded similar results, but the posterior model probabilities from FFX appeared over-confident. This observation reflects the fact that the RFX inference properly accommodates between-subject variability, whereas FFX does not.

Another important point is the behaviour of the method in the presence of outliers. Since the RFX approach takes into account group heterogeneity, it has proven (Stephan et al., 2009) to be more robust than FFX. In our fMRI analysis, we have confirmed this result. Moreover, we have observed that the two analyses yield contradictory results for brain regions where one of the subjects provides strong evidence in favour of one particular model, contrary to the rest of the subjects. The results from FFX are adversely influenced by this single subject, whereas the RFX inference was not.

A minor disadvantage of our new approach is that it relies on the prior computation of log-evidence maps for each subject and model. These computations are more time consuming than the standard statistical parametric mapping approach by a factor of five to ten. However, these individual subject maps need only be computed once for all subsequent group BMS analyses. The method proposed here for constructing BMS maps is not so computationally demanding and takes on average less than half an hour to create whole-brain PPMs for the comparison between two models using the log-evidence images from 12 subjects on a standard PC. Moreover, we envisage that our new approach may be most usefully applied to regions or networks of regions previously identified using functional localiser methods. The use of these localisers has the advantage of speeding up the computation and reducing its time to approximately less than a minute for a region with a few thousand voxels.

In the current work, log-evidence maps were smoothed by a user-specified FWHM Gaussian kernel. This will be finessed in future work to include a spatial model over *r* and its smoothness estimated using a novel Bayesian framework. This would mirror corresponding developments in the analysis of group data from M/EEG source reconstructions (Litvak and Friston, 2008).

The product of the analysis procedures described in this paper are posterior probability maps. These show voxels where the posterior probability over model frequency exceeds some user-specified value. In a previous work (Friston and Penny, 2003), we have derived PPMs over effect size. We note that, as is common-place in Bayesian inference, these posterior inferences could be augmented with the use of decision theory. This requires the costs of false negative and false-positive decisions to be specified. One can then use decision theory to make decisions which minimise, for example, the posterior expected loss (Gelman et al., 1995). In addition, we note a connection between posterior probabilities and false discovery rate, in which if above threshold values are declared as activations, a posterior probability of greater than 95% implies a rate of false discoveries less than 5% (Friston and Penny, 2003). It is also possible to relate posterior probabilities to the realised false discovery rate (rather than an upper bound or the expected FDR) (Muller et al., 2007). Finally, we note that a comprehensive Bayesian thresholding approach has been implemented by Woolrich et al. (2005). This work uses explicit models of the null and alternative hypotheses based on Gaussian and Gamma variates. This requires a further computationally expensive stage of model fitting, based on spatially regularised discrete Markov random fields, but has the benefit that false-positive and true-positive rates can be controlled explicitly.

Unlike classical inference using *F*-tests, our framework allows for comparison of non-nested models, which we hypothesize will be useful in a number of experimental domains. One such domain is model-based fMRI (O'Doherty et al., 2007) in which computational models are first fitted to behavioural data, and sets of regressors derived to be used as predictors of brain imaging data. A typical example is the study of behavioural control using computational models and fMRI (Montague et al., 2004). The use of model comparison maps in addition to model-based fMRI would allow brain imaging data to directly adjudicate, for example, between different computation models of value updating (Montague et al., 2004). In this paper, we have compared information theoretic models of novelty processing, and this will continue to be the subject of future publications.

## Software note

The algorithms described in this note have been incorporated into the current version of the SPM software (SPM8, http://www.fil.ion.ucl.ac.uk/spm/). Bayesian model selection can be implemented and the results visualised via the user interface (Stats > Bayesian Model Selection > BMS: Maps). This calls lower-level routines such as the random effects model selection function, ‘spm_bms’.
